# Brain Responses to Emotional Faces in Natural Settings: A Wireless Mobile EEG Recording Study

**DOI:** 10.3389/fpsyg.2018.02003

**Published:** 2018-10-25

**Authors:** Vicente Soto, John Tyson-Carr, Katerina Kokmotou, Hannah Roberts, Stephanie Cook, Nicholas Fallon, Timo Giesbrecht, Andrej Stancak

**Affiliations:** ^1^Department of Psychological Sciences, University of Liverpool, Liverpool, United Kingdom; ^2^Institute for Risk and Uncertainty, University of Liverpool, Liverpool, United Kingdom; ^3^Unilever Research & Development Port Sunlight Laboratory, Merseyside, United Kingdom

**Keywords:** EEG, eye-movement related potentials, N170 component, source dipole analysis, MoBI, mobile brain imaging, visual evoked potential (VEP)

## Abstract

The detection of a human face in a visual field and correct reading of emotional expression of faces are important elements in everyday social interactions, decision making and emotional responses. Although brain correlates of face processing have been established in previous fMRI and electroencephalography (EEG)/MEG studies, little is known about how the brain representation of faces and emotional expressions of faces in freely moving humans. The present study aimed to detect brain electrical potentials that occur during the viewing of human faces in natural settings. 64-channel wireless EEG and eye-tracking data were recorded in 19 participants while they moved in a mock art gallery and stopped at times to evaluate pictures hung on the walls. Positive, negative and neutral valence pictures of objects and human faces were displayed. The time instants in which pictures first occurred in the visual field were identified in eye-tracking data and used to reconstruct the triggers in continuous EEG data after synchronizing the time axes of the EEG and eye-tracking device. EEG data showed a clear face-related event-related potential (ERP) in the latency interval ranging from 165 to 210 ms (N170); this component was not seen whilst participants were viewing non-living objects. The face ERP component was stronger during viewing disgusted compared to neutral faces. Source dipole analysis revealed an equivalent current dipole in the right fusiform gyrus (BA37) accounting for N170 potential. Our study demonstrates for the first time the possibility of recording brain responses to human faces and emotional expressions in natural settings. This finding opens new possibilities for clinical, developmental, social, forensic, or marketing research in which information about face processing is of importance.

## Introduction

Facial expressions are evolutionarily based and culturally conditioned tools. They steer social interactions, solicit help and inform about events in social environments as well as the intentions of the expresser (Matsumoto et al., 2008). The capacity to recognize facial expressions quickly and correctly correlates with problem solving capacity and efficient adaptation to a new environment (Matsumoto et al., 2004). In contrast, the ability to recognize facial expressions is impaired in abused children (Camras et al., 1988), depressed people (Persad and Polivy, 1993), children presenting autistic traits (Ozonoff et al., 1990), and people with a history of substance abuse (Foisy et al., 2005).

Previous brain imaging studies have shown that a set of brain regions in occipitotemporal cortex were associated with processing human faces (Kanwisher et al., 1997; Haxby et al., 2002). Electroencephalographic event-related potentials (ERPs) revealed a negative potential, N170, at lateral occipitotemporal regions of the scalp which responded with greater amplitude when viewing faces compared to objects (Bentin et al., 1996). A large number of studies have confirmed that the N170 not only reflects low-level visual features of a human face, but would also signify a conscious awareness of the presence of face in the visual field [reviewed recently in (Rossion, 2014; Olivares et al., 2015)]. While earlier studies reported a lack of encoding of emotional facial expression by the N170 potential (Herrmann et al., 2002; Eimer et al., 2003), a recent meta-analysis confirmed the encoding of emotional facial expressions in the amplitudes of the N170 potential (Hinojosa et al., 2015).

Human perception and cognition in real life differs from that occurring in a laboratory experiment in that it offers a continuous and naturally flowing stream of perceptual and motor decisions. Unlike flashing a visual stimulus on a screen in a laboratory experiment, free viewing of scenes under natural conditions involves multi- and *trans*-saccadic processes which necessitate the anticipation of a visual pattern before the start of a saccadic eye movement, and integration of information across successive eye fixations (Melcher and Colby, 2008). In contrast to laboratory electroencephalography (EEG), magnetoencephalography (MEG) or functional magnetic resonance imaging (fMRI) experiments, people often interact with real life situations while walking or standing upright. Maintaining an upright stance or walking poses further demands on the brain processing and physiological adjustments which are not encountered in laboratory settings (reviewed in Thibault et al., 2014). Visual processing is enhanced and electrical activity and the relative cerebral blood flow in occipital cortex (Goodenough et al., 1981; Ouchi et al., 2001) is enhanced while standing erect compared to reclining. Therefore, brain responses to viewing human faces, which have been well established in a number of laboratory studies over past decades, cannot be taken as templates which the brain merely replays in a real life situation such as walking and meeting people.

Recent advances in EEG technology and data analysis opened new possibilities to explore human cognition, emotion and actions as they occur in natural settings. A novel non-invasive mobile brain and body imaging (MoBI) modality has been proposed (Makeig et al., 2009; Gramann et al., 2010, 2011). MoBI typically involves the use of wireless EEG recordings in freely moving individuals, and a multimodal approach to data analysis which combines EEG recordings with recordings of muscle activity, spatial head coordinates, and electro-oculography (Ojeda et al., 2014). The challenges posed by recording wireless EEG in natural settings are largely related to the presence of movement related artifacts and the separation of cerebral and extracerebral sources of EEG activity (Gwin et al., 2010). A MoBI approach has been successfully applied to recording of EEG during every-day life activities (Wascher et al., 2014) like cycling (Zink et al., 2016), recording of EEG in pilots while airborne (Callan et al., 2015), and identification of brain potentials related to the control of locomotion (Severens et al., 2012; Wagner et al., 2012; Seeber et al., 2015).

Recording brain electrical potentials during viewing human faces in natural settings poses an additional specific challenge related to the absence of a time locking event in a continuous stream of EEG data, which has traditionally been provided by a stimulus control computer. In the present study, we employed continuous recordings of eye movements to identify the time instants at which the gaze first landed on a picture of a face or object. This approach capitalizes on previous laboratory studies analyzing eye-movement related potentials during free reading of words (Baccino and Manunta, 2005; Hutzler et al., 2007; Dimigen et al., 2011) or free viewing of visual scenes (Fischer et al., 2013; Simola et al., 2015) and is similar to a recent study which reconstructed the face N170 potential during viewing of a continuous video stream (Johnston et al., 2014).

The primary aim of this study was to employ a mobile brain and body imaging approach to record face-specific brain potentials in freely moving individuals. As a secondary goal, we also analyzed whether hedonic valence of faces and objects would manifest in mobile EEG data. In line with previous research studies conducted in laboratory settings (Kolassa and Miltner, 2006; Blau et al., 2007; Trautmann-Lengsfeld et al., 2013), we hypothesized that face-specific brain activations will manifest in the right occipitotemporal region of the scalp at about 180 ms, and that the face-sensitive eye movement related potential (EMRP) component will be modulated by the emotional expressions of the faces. Pictures of objects and face expressions consisted of pleasant, neutral, or unpleasant to explore the possibilities of mobile EEG recordings to both differentiate brain responses to faces and objects and to resolve the qualities of emotional expression of faces.

## Materials and Methods

### Participants

Twenty-five healthy volunteers (27.2 ± 4.7 years old, mean ± SD) were recruited for the study. A total of six participants were excluded from the sample due to signal problems either in the eye tracking (*n* = 4) or wireless EEG recording (*n* = 2). Thus, the final sample consisted of nineteen participants (five females) with an average age of 27 ± 5 years. All participants gave their written informed consent prior to the study. Ethical approval was obtained from the University of Liverpool Research Ethics Committee. Participants received £20 as compensation for their travel expenses and time.

### Stimuli

Stimuli consisted of 180 color pictures of face and non-face objects. Object stimuli included toys, flowers and gifts (positive), dirty toilets, rubbish bins and scenes of contamination (negative), and houses, stationary and household objects (neutral). Complex images such as landscapes with flowers and scenes of contamination were included in the object images to enable modulation of the emotional valence of the object category. The luminance levels of face and objects in each of three levels of hedonic valence were similar and not statistically different (*P* > 0.05). Ninety face pictures were selected from the NimStim Set of Facial Expressions (Tottenham et al., 2009) and 90 object pictures were taken from the IAPS database (Lang et al., 1997) and from public domain images available under creative commons licenses. In each category (objects and faces), 30 hedonically positive, 30 negative, and 30 neutral pictures were selected. Face stimuli were comprised of happy (positive), neutral and disgusted (negative) facial expressions. The expression of disgust was chosen because this emotion best matched the emotional response to unpleasant object images. All face images showed a frontal view on a white background. Seventeen different female faces (9 White/Caucasian, 4 East-Asian and 4 Afro-Caribbean) and eighteen male (10 White/Caucasian, 6 Afro-Caribbean and 2 East-Asian) faces were used in the study.

**FIGURE 1:**
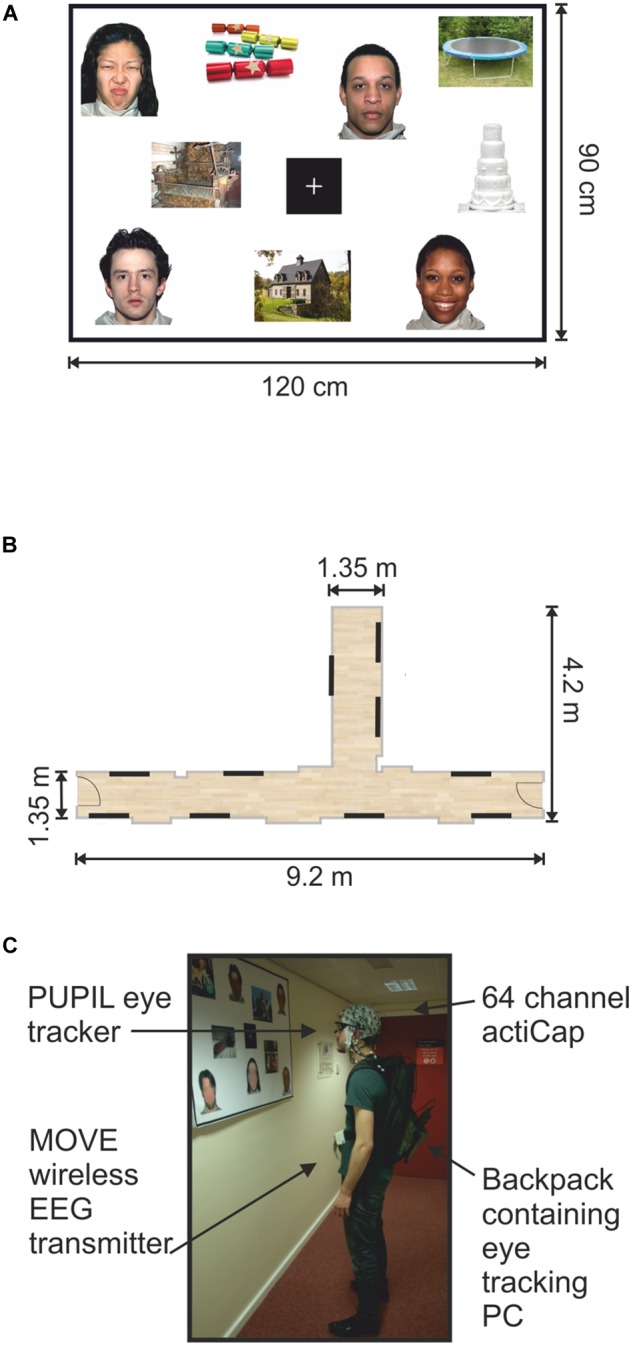
Mock gallery and wireless EEG recordings. **(A)** Example of one poster (120 cm × 90 cm) containing nine pictures and a fixation cross. **(B)** Schematic illustration of the hallways used to set up the mock gallery. Black lines indicate the locations of the 10 panels throughout the mock gallery. **(C)** One participant viewing images while wearing the wireless EEG and a portable eye-tracker. The backpack held the laptop computer recording the eye-tracking. The man appearing in **(C)** is one of the authors of this work and provided informed written consent to appear in the image.

Stimuli were presented on twenty A0 poster-size panels. Each panel contained 9 images (15 cm × 20 cm) and a fixation cross in the center (14.3 cm × 14.3 cm) printed onto a paper sheet (Figure 1A). All images were assigned pseudo-randomly to present a minimum of three faces and three objects (one of each hedonic category) per panel. The face and object pictures were distributed quasi-randomly on each poster in such a way that no face picture would systematically fall into the visual field while shifting the gaze to a face picture in the corner of a panel.

All panels were pasted onto Styrofoam sheets and attached to the walls using adhesive tape. Two hallways within the Eleanor Rathbone Building of the University of Liverpool were used to create a mock art gallery where the experiment took place (Figure 1B).

### Procedure

Our study is an initial attempt to record and quantify the face-sensitive ERPs that occur in natural settings such as in the street or at the supermarket. The experiment closely matches the natural settings of a picture gallery in which individuals move freely from one painting to another and visually explore a painting containing both human figures and non-living objects. The presence of the fixation cross in the middle of the board which participants fixated before shifting their gaze to a next picture on the board was the only difference relative to the settings of a picture gallery. This component was introduced to the task to reduce the possibility of overlap in viewing neighboring pictures and to compensate for the limited capacity of our eye-tracker to quantify the pattern of saccades and fixations during a free visual exploration which would be required to reconstruct the eye-movement related potentials using advanced processing methods such as regression analysis (Ehinger and Dimigen, 2018).

A mock art gallery was created by hanging the stimuli panels onto bare walls in designated hallways. The corridors were not closed off on either side, nor were there attempts to discourage other people from passing through while an experimental session was in progress. As in the real world, passers-by occurred spontaneously. Participants were requested to walk through the mock art gallery while viewing the images displayed on each panel. They were free to navigate the gallery in any order they chose and view individual pictures in any order and for as long as they wished. Subjects were only instructed to stand facing each panel and to view each image for at least few seconds before moving onto the next image. Participants were, additionally asked to look at the center fixation cross before viewing each image, and to return their gaze to the fixation cross before moving to the next picture. They only continued onto a subsequent panel after viewing all images (Figure 1A). Picture preferences were indicated by marking selected pictures on a small paper printed version of each panel.

The gallery task was divided into two blocks. Each block contained ten panels with nine images presented on each one. In total, participants viewed 180 different images in the experiment. On average, each gallery block lasted approximately 15 min. Participants were tasked with selecting a preferred face and object as well as a disfavored face and object from each panel.

Instructions were delivered and equipment was set up in a designated lab space. Participants were fitted with the EEG cap (actiCAP, Brain Products, Germany). The mobile EEG system was then connected and wireless signals were visually inspected on a standing participant. Next, eye tracking glasses (PUPIL; Kassner et al., 2014) were placed on the participant over the EEG cap. The eye trackers were calibrated against a blank white panel at a distance of 1 m. Gaze-tracking was optimized by means of 3D calibration routine using manual markers.

The eye tracking recording laptop was placed in a backpack and carried by the participant for the duration of the gallery task (Figure 1C). EEG cables running from the electrodes to the lightweight transmitter were also placed in the backpack to reduce cable sway artifacts (Gramann et al., 2010; Gwin et al., 2010). A mobile base unit was assembled using a rolling trolley where the wireless signal receiver, EEG amplifier and recording computer were placed. The base unit was positioned by the experimenter maintaining a distance of no more than 7 m from the participant in order to maintain optimal signal quality.

Electrode impedances and gaze tracking calibration were checked and corrected in the break between blocks if required. Once the gallery task concluded, subjects completed a rating task on a computer in the laboratory.

Once the gallery task had been completed, the EEG cap and the eye tracking glasses were removed. Participants were then required to rate how much they liked and if they would approach the images previously seen in the mock gallery. Ratings were performed using two visual analog scales (VAS) sized 10 cm and anchored on each extreme (i.e., ‘*0: Do not like’* up to *‘100: Like very much’* and ‘*0: Avoid’* up to *‘100: Approach’*). Pictures and rating scales were presented on a LCD screen using Cogent program v. 1.32 (Welcome Department of Imaging Neuroscience, United Kingdom) running on MATLAB v. R2014a (The MathWorks, Inc., United States).

### EEG Recordings

Whole scalp EEG data was continuously recorded using a 64-channel wireless and portable EEG system (Brain Products, GmbH, Münich, Germany). Signals were digitized at 1 kHz on a BrainAmp DC amplifier linked to Brain Vision Recorder program v. 1.20.0601 running on a Windows laptop. The wireless interface (MOVE, Brain Products, GmbH) utilizes a lightweight signal transmitter which participants carry on a belt (Figure 1C). Active Ag/AgCl EEG electrodes were mounted on an electrode cap (actiCAP, Brain Products, GmbH) according to the 10–20 electrode system. Electrode FCz was used as the system ground and electrodes were referenced to Cz. The EEG cap was aligned in respect to the midpoint between the anatomical landmarks of the nasion and inion, and the left and right preauricular points. The electrode-to-skin impedances were lowered using electrolyte gel (Signa Gel, Parker Laboratories, Inc., Fairfield, NJ, United States) and checked to be below 50 kΩ before starting the recordings.

### Eye Movement Recording and Analysis

The locations of the gaze positions were recorded using a PUPIL binocular eye-tracking system and Pupil Capture software v. 0.7.6. running on Ubuntu v. 14.04.4. The PUPIL eye tracker is a high resolution lightweight wearable system (Figure 1C). PUPIL software is a cross-platform (Linux, Mac, and Windows) open-source software which is actively maintained and supported by the developers (Kassner et al., 2014). Here, eye-tracking data and the real-world video streams were set at a sampling rate of 60 frames per second with a resolution of 600 pixels × 800 pixels in both the world camera and in the eye cameras. The sampling rate of 60 Hz was chosen based on pilot experiments in order to secure a continuous stream of eye-tracking data which was often discontinuous at higher sampling rates.

The ocular pupils of both eyes were located based on a center-surround detection algorithm (Swirski et al., 2012). To calibrate the gaze locations, a manual marker 3D calibration protocol was used to generate a 9-point grid in the field of view of the participant. Calibration was repeated until gaze positions were accurate everywhere on the blank panel. Small calibration offsets occurred at times due to displacements in the wearable eye-tracker on the subject’s face. These were adjusted using the manual gaze correction plug-in on Pupil Player during manual tabulation of stimulus onset times. If multiple time stamps were associated with one frame in the real-world recordings, the middle frame was selected as the time-locking event. The Pupil Capture software can process up to three video streams (two eye cameras and a world view camera) synchronously and allows for mid-recording calibrations. These video streams are read and exported using Pupil Capture software for real-time pupil detection, recording, and gaze mapping. The gaze mapping function allows the eye positions to be superimposed onto the world-view scene space. Exported PUPIL raw gaze data is time-locked to the processing computer’s internal clock, giving millisecond precision to the eye measurements.

Eye-tracking data were processed using Pupil Player v. 0.7.6 program. Additionally, all recorded frames contained an accurate time stamp based on the PC processor real-time clock. Eye tracking video files were visually inspected and stimuli onsets were manually tabulated. Each stimulus was logged on a picture by picture basis with stimulus onset defined as the first instance in which the gaze position touched or landed on an image. The real times corresponding to the tabulated frames were used to import stimulus onset latencies onto the raw EEG data. Four subjects’ data was excluded from the sample due to loss of gaze calibration during the recordings.

The total gaze times were calculated by additionally tabulating the earliest frame in which the gaze left an image. Of the 19 total subjects, 15 data sets were used to calculate the average gaze times per condition. Four subjects’ data was not included in the calculation of mean viewing times. The four exclusions were due to difficulties or uncertainties in defining an accurate offset time when the subject’s gaze left an image to return to the fixation cross. A 2 × 3 ANOVA for repeated measures was used to check any significant differences in viewing times across conditions.

At the start of each gallery block, a trigger-box fitted with a light emitting diode (LED) was used to synchronize data streams. A pulse of light was flashed into the world-view camera on the eye trackers as a simultaneous transistor–transistor logic (TTL) pulse was registered in the EEG data recording. In doing so, a visual light cue became apparent at a specific frame in the eye tracking video data. This frame was then registered and used to temporally synchronize the EEG and eye-tracking data streams. The accuracy of the synchronization was tested in a 15 min recording during which 15 synchronizing light stimuli were produced in approximately 1 min intervals. The time-locked eye tracking frames were logged and compared to the latency of the EEG triggers. The temporal asynchrony between triggers simultaneously delivered to the eye tracking and EEG recording system was of 0.022 ± 0.020 s (mean ± SD) over a 15 min recording.

### Eye Movement Related Potentials

After synchronization, event markers were inserted into EEG data by synchronizing the time axes of the EEG and eye-tracking system. EEG data were pre-processed using the Brain Electrical Source Analysis program (BESA v.6.1, MEGIS Software GmbH, Munich, Germany). Data were first referenced to a common average using common averaging method (Lehmann, 1987) on the continuous EEG signal. EEG data were epoched to range from -200 to 1000 ms and the mean EEG activity in the baseline interval ranging from -200 to -100 ms was removed from each data point. The onset of a stimulus was defined as the first contact of the gaze with any part of a picture in each of the 180 images. This time point effectively corresponded to part of the saccade which brought the gaze onto a particular face or object in a picture. Eye blink artifacts were removed using a pattern matching algorithm involving principal component analysis (Berg and Scherg, 1994; Ille et al., 2002). Then, EEG data was visually inspected for movement or muscle artifacts and trials contaminated with large artifacts were marked and excluded from further analysis. Post-saccadic EMRPs were computed from all trials falling into six different conditions (face vs. objects, three hedonic levels).

The data was visually inspected and corrected for the presence of artifacts. Trials were excluded if artifacts were present in either eye-tracking or EEG data. If participants skipped an image, failed to fixate, or gaze tracking was lost during fixation, the concurrent trial was discarded.

The sampling rate of the eye-tracking device was calculated offline (41.1 Hz on average). Given the relatively low sampling rate, we have not analyzed in detail if the next eye movement was a saccade or a fixation. Thus, EEG epochs were formed as cuts into a wild video scene similar to a recent study (Johnston et al., 2014).

### Source Dipole Analysis

As EEG epochs were effectively locked to the first shift of the gaze onto a picture, we anticipated that EMRPs will comprise the saccade-related cortical potential (Yagi, 1981a; Thickbroom et al., 1991; Kazai and Yagi, 2003) and artifact potential components related to offsets of saccades. The eye movement artifacts primarily involve the corneoretinal potential associated with a displacement of the large electrical dipole of the eye during eye blinks or saccades, and a smaller saccade spike potential related to the contraction of oculomotor muscles at onset of a saccade (Dimigen et al., 2011; Nikolaev et al., 2016). Owing to limited sampling rate of eye-tracking data and presence of a small jitter between eye movement and EEG data, we applied source dipole modeling to separate electrical activations originating in the occipitotemporal cortex from those electrical potentials which originated in eye orbits and were volume conducted to distant regions of the scalp.

Grand average EMRP waveforms were used to determine the source dipole locations. A source dipole model was constructed using BESA v. 6.1 program. Two regional sources were used to model the electrical potentials to the residual corneoretinal artifact and saccade spike potentials (Berg and Scherg, 1991). The lambda component is an occipital potential that becomes most prominent when averaged EEG signals are time-locked with a saccadic eye movement offsets. To model the cortical sources accounting for distinct peaks of lambda potential (Yagi, 1979; Thickbroom et al., 1991), a set of equivalent current dipoles (ECD) were fitted using a sequential strategy (Stancak et al., 2002; Hoechstetter et al., 2010). In sequential strategy, ECDs are successively fitted based on the peak latencies of the prominent ERP peaks determined in the global field power curve. Each new ECD explains the portion of data variance not explained by previously fitted ECDs. First, we placed two regional sources into the right and left eye orbit to separate any corneoretinal potentials related to eye blinks or saccades from the cerebral sources which were modeled in the next stage. A regional source has three orthogonal dipoles with origins at the same location and can therefore model potentials emanating from one location in all possible directions. Since regional sources have three orthogonal components, they model activations from a widespread region of the scalp and activations which do not have constant orientations over the entire EMRP epoch. While placing regional sources into eye orbits is less effective than modeling saccade potentials with a set of equivalent dipoles each tuned to a specific saccade direction (Berg and Scherg, 1991), this method was an appropriate choice in the absence of information about the timing and angles of saccades.

We added another dipole with free orientation and location to the source dipole model. Fitting this additional dipole resulted in the dipole being placed beyond the boundaries of the head and not changing the residual variance which means that the extra dipole did not explain any specific topographic aspect of the potential field. Secondly, we also modeled the potentials in the time points of interest using a classical LORETA (Pascual-Marqui et al., 1994) analysis recursively applied (CLARA) (Hoechstetter et al., 2010). CLARA did not show any new cluster beyond the locations previously tagged by equivalent current dipoles (ECD).

The CLARA analysis was also used to verify the locations of ECDs using an independent source localization method (Hoechstetter et al., 2010). A 4-shell ellipsoid head volume conductor model was employed to construct the source dipole model using the following conductivities: brain = 0.33 S/m; scalp = 0.33 S/m; bone = 0.0042 S/m; cerebrospinal fluid (CSF) = 1.0 S/m. Finally, we have compared the scalp potential maps with the potential maps predicted by the source dipole model and found a good match.

Due to the limited sampling rate of the eye tracker, presence of a small jitter between the EEG and eye tracking data streams, and absence of calibration marks in visual scenes, we were not able to identify individual saccades and evaluate their impact on EEG potentials in the present study. Indeed, the principal orthogonal component in each of two regional sources shows the presence of an eye movement potential starting about 20 ms before the time-locking event and continuing eye-movement potentials after the time-locking event. This variability is due to the triggering of stimulus onset which was determined as the first instance of the gaze contacting an image. Due to the position of the images on the panels relative to the fixation cross, this occurred at different instances of the saccade. Evaluation of effects of including two regional sources on residual variance is given in the Section “Results.”

### Statistical Analysis

The source waveforms representing the source activity in each of the fitted ECDs were analyzed using a 2 × 3 repeated measures ANOVA (objects vs. faces, three levels of hedonic valence). To control for the risk of false positive results due to a large number of tests, *P*-values were corrected using the false discovery rate method (Benjamini and Yekutieli, 2001). This analysis was used to identify the latency interval in which faces and objects and/or three hedonic categories would differ. Average source activity in intervals of interest was analyzed further in SPSS v.22 (IBM Corp., Armonk, NY, United States). *Post hoc* paired *t*-tests were performed and considered significant at *P* < 0.05. *P*-values reported hereafter are corrected for multiple comparisons when necessary.

Scalp data at select electrodes were analyzed similarly as source dipole waveforms. The ANOVA of the raw data was computed for the average of P8 and PO8 electrodes on the scalp. This analysis was included to enable comparisons of EMRP with previous studies.

## Results

### Behavioral Ratings

**FIGURE 2:**
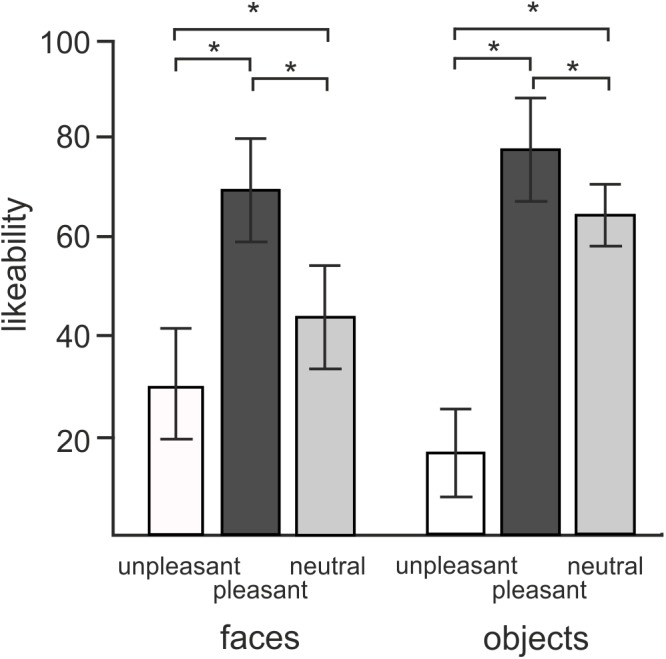
Mean ratings of likeability for objects and faces in three different hedonic valence conditions. Error bars stand for standard deviations. Asterisks (^∗^) indicate the presence of statistical significance at *P* < 0.05.

Figure 2 illustrates the average liking scores for each of the experimental conditions. A 2 × 3 repeated measures ANOVA revealed a statistically significant effect of objects vs. faces likeability [*F*(1,18) = 7.7, *P* = 0.012]. Subjects attributed larger likeability to objects (52.6 ± 4.25, mean ± SD) than faces (47.5 ± 7.53). Both objects and faces showed a statistically significant effect of hedonic valence [*F*(2,36) = 179.4, *P* < 0.0001] consisting of a greater likeability of both objects and faces of positive valence compared to both neutral and negative valence, and greater likeability of neutral than negative valence (*P* = 0.012). The interaction between objects vs. faces and three hedonic categories was also statistically significant [*F*(2,36) = 62.1, *P* < 0.0001]. *Post hoc* tests revealed that this interaction effect was driven by a greater contrast between neutral and unpleasant objects [*t*(18) = -17.4, *P* < 0.0001] than neutral and unpleasant faces [*t*(18) = 5.8, *P* < 0.0001].

A 2 × 3 repeated measures ANOVA conducted on average approachability ratings showed a similar pattern of responses. Subjects rated objects (53.2 ± 0.98) as more approachable than faces (44.5 ± 1.6) [*F*(1,18) = 19.8, *P* < 0.0001] as well as rating positive pictures (70.6 ± 2.3) more approachable than neutral (52.4 ± 1.4) or unpleasant ones (23.56 ± 1.8) [*F*(1,16) = 140.5, *P* < 0.0001]. *Post hoc* comparisons showed an interaction effect with greater contrasts shown between neutral and negative valence objects [*t*(18) = -13.8, *P* < 0.0001] relative to the contrast between neutral and disgusted faces [*t*(18) = -6.7, *P* < 0.0001] [*F*(2,36) = 44.4, *P* < 0.0001].

The 2 × 3 ANOVA for repeated measures was performed on the average gaze time of each subject across conditions. The data from 15 subjects was used in this analysis. The four exclusions from this analysis were due to difficulties in defining an accurate offset time when the subjects gaze returned to the fixation cross. No effects of category [*F*(1,14) = 2.11, *P* > 0.05] were found in mean viewing times between face (3.346 s ± 1.3 s) and object (3.155 s ± 1.1 s) images. Nor were there any effects of hedonic valence found on the average viewing times either [*F*(1,14) = 0.451; *P* > 0.1]. Disgusted (3.15 s ± 1.1 s), neutral (3.28 s ± 1.3 s), and happy faces (3.31 s ± 1.3 s) were viewed equally across face and object categories (*P* > 0.1).

### Eye Movement Related Potentials

Wireless EEG data maintained good quality throughout the duration of the experiment. As subjects maintained a stable stance during the viewing of pictures, EEG data showed minimal neck muscle or head movement artifacts which have been shown to heavily affect EEG data during walking or running (Gwin et al., 2011). Of the 6 excluded subjects, 2 of these were discarded at an early stage in the experiment due to loss of signal purportedly caused by drainage of the batteries in the transmitter unit resulting in incomplete EEG data. The average number of accepted trials was 72 ± 8.6 and 73 ± 6.3 (mean ± SD) for face and object pictures, respectively.

**FIGURE 3:**
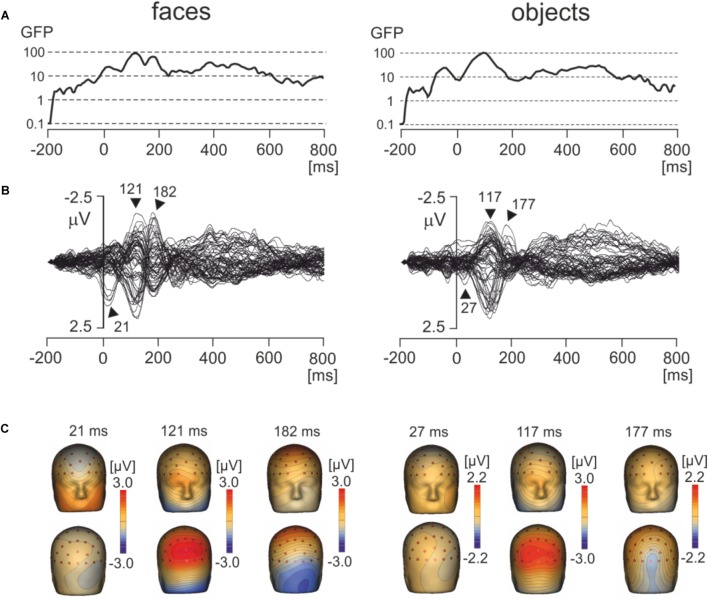
Grand average EMRPs during viewing of faces and objects. **(A)** Global field power for face and object pictures. **(B)** Butterfly plots of grand average EMRPs to face and object pictures. Peak latencies of distinct EMRPs components are highlighted with arrows. **(C)** The topographic maps of grand average EMRPs overlaid on the volume rendering of the human head at select latency points.

Figure 3A shows the global field power and Figure 3B the butterfly plots of EMRPs for face and objects. Figure 3C illustrates the topographic maps of distinct EMRP components observed in global field power curves. An early small potential component peaking at 27 ms in objects and at 21 ms in faces was associated with a weak negative potential in the right occipital region of the scalp and another weak negative potential at the vertex (Figures 3B,C). It is unclear whether this small potential was a part of an anticipation of a face picture or whether it was related to the effects of saccades preceding the time-locking event. The lambda potential (Yagi, 1981a; Thickbroom et al., 1991) showed a large component peaking at 117 ms in objects and at 121 ms in faces and exhibited a prominent positive component at occipital electrodes. The peak latency of the lambda potential suggests that the time-locking event coincided more often with onsets of saccades rather than with onsets of fixations because the latencies of the lambda potential occur comparatively late (∼120 ms) if the time locking event is the saccade onset (Kaunitz et al., 2014). Only in face EMRPs, a distinct negative component peaking at 182 ms was seen in the right occipital-temporal region of the scalp. This negative component was associated with a positive potential component at central-parietal electrodes (Figure 3C). Both the peak latency of the negative occipitotemporal component and the presence of a positive vertex potential suggest that this particular component, responding only to face pictures, was the face-sensitive N170 component (Bentin et al., 1996). The butterfly plots for both faces and objects showed the presence of electrical activity in the latency epoch >300 ms. However, these components had relatively small amplitudes compared to the earlier latency components and did not show distinct peaks allowing further detailed analysis.

### Source Dipole Analysis

**FIGURE 4:**
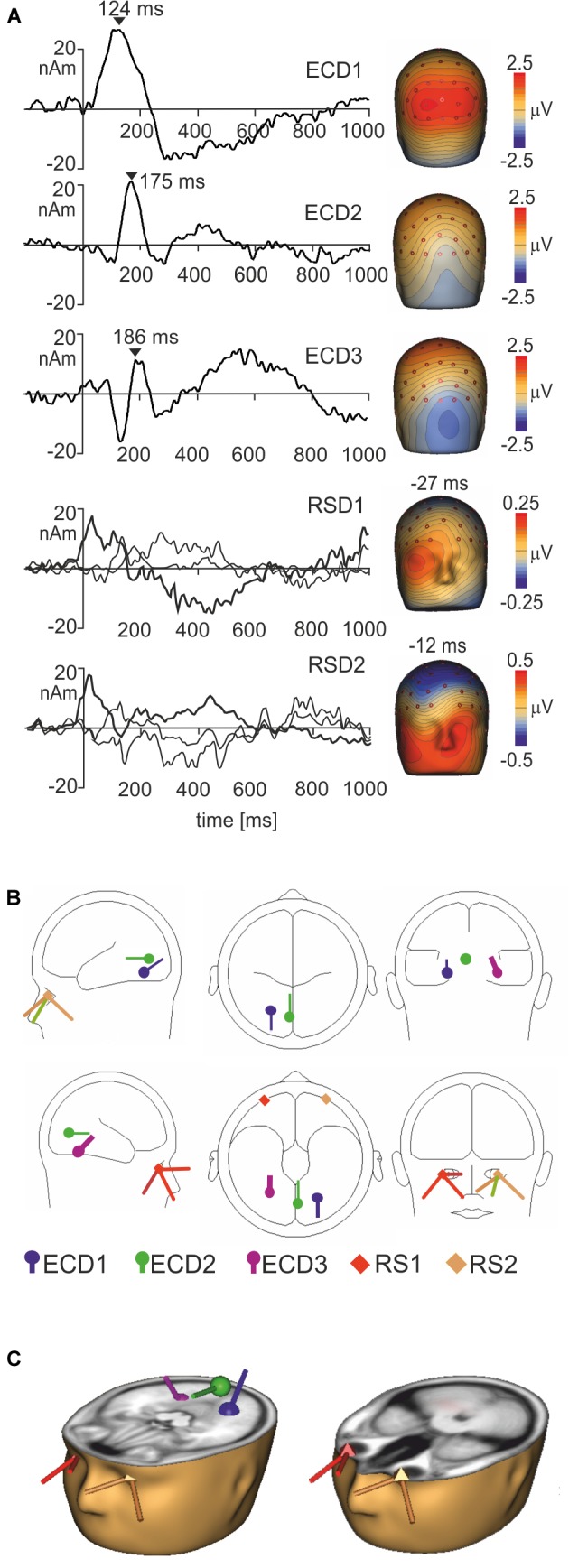
Source dipole model of grand average face and object EMRPs. **(A)** The left panel shows the source dipole waveforms of three ECDs (ECD1–ECD3) and two regional sources (RS1-2). The right panel illustrates the spatial topographic maps at the latency points showing the strongest source activity (ECD1–ECD3) or at latency points showing a spatio-temporal pattern of corneoretinal potential (RS1-RS2). **(B)** The glass brain showing locations and orientations of ECD1–ECD3 and RSD1-2. **(C)** Locations and orientations of ECDs and RSDs in the standardized MR of a human head. The left head shows three ECDs, and the right head illustrates the two regional sources located in both eye orbits.

To segregate brain electrical responses generated in localized cortical regions from the extracerebral potentials, EMRP data were analyzed at the source dipole level. The source dipole model was built using grand average EMRPs comprising data from six conditions (faces and objects, three hedonic valence categories) and all subjects. Figure 4A illustrates the source dipole waveforms and spatial topographic maps of EMRP waveforms for each fitted source dipole. Figure 4B illustrates the source dipole locations and orientations in a schematic transparent glass brain. Figure 4C demonstrates the locations of individual source dipoles in an anatomical brain image as well as the two regional sources in the eyes.

It should be pointed out that the use of regional sources has a drawback of having the sources just outside of the head model (Lins et al., 1993) potentially causing incomplete removal of saccadic potentials occurring during free viewing of pictures. The regional sources showed distinct peaks related to saccade offsets, and further irregular waves related to eye movements occurring later. Placing two regional sources into the eye orbits was an additional precaution, on top of the removal of eye blink artifacts from raw data using the pattern matching algorithm, in preventing the extracerebral sources from affecting the EMRPs. Nevertheless, dipole locations results must be taken with some caution due to the difficulties associated with generating precise estimations of source locations in mobile EEG data which can, at times, be noisy (Grech et al., 2008).

ECD 1 was fitted to the visual cortex (Brodmann area 19; approximate Talairach coordinates: *x* = -22.5, *y* = -65.4, *z* = -18.2 mm) (Figures 4B,C) and modeled the large positive component of the lambda potential. It peaked at 124 ms and accounted for the positive potential maximum in occipital and lower parietal electrodes. ECD2 was located in the primary visual cortex (Brodmann area 17; approximate Talairach coordinates: *x* = -3.5, *y* = -74.6, *z* = 2.7 mm) (Figures 4B,C). This source accounted for a negative potential occurring briefly at 175 ms in occipital electrodes. This potential was mainly featured in objects EMRPs.

ECD3 accounted for the N182 potential showing a negative maximum in the right temporal-occipital electrodes and a positive potential maximum over the central and parietal regions of the scalp. This spatio-temporal pattern was prominent in face picture data and almost absent in objects data and therefore, the final fit of ECD3 was carried out in face EMRPs. The source of this potential component was located in the right fusiform gyrus (Brodmann area 37; approximate Talairach coordinates: *x* = 25.7, *y* = -56.8, *z* = -18.2 mm) (Figures 4B,C).

**FIGURE 5:**
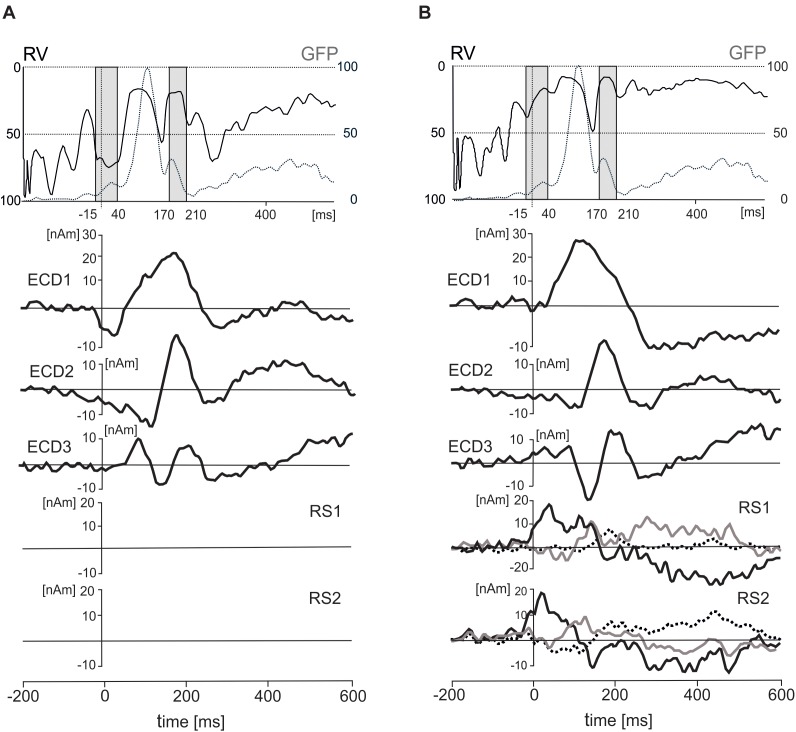
Effects of inclusion of two regional sources (RS1 and RS2) on residual variance (RV), standardized global field power (GFP), and three equivalent current dipoles (ECD1–ECD3). **(A)** The time courses of RV (solid line), GFP (dotted line), and three ECDs after excluding the two RSs. **(B)** The time courses of RV, GFP, three ECDs and two RSs in a solution with the regional sources included. The two gray intervals in the top panel correspond to two intervals of interest in which residual variance was evaluated statistically (–15 – 40 ms and 170 – 210 ms). The dot lines in both rectangles stand for the latency of 0 ms. The principal of three orthogonal components in each regional source is plotted with black bold line.

While eye tracking data provided a useful trigger for computing the event-related potentials in the present study, the limited sampling rate and the lack of precise, calibrated markers in spontaneously occurring visual scenes did not allow quantification of oculo-motor artifacts similar to previous studies conducted in laboratory settings (Ossandón et al., 2010; Rämä and Baccino, 2010; Dimigen et al., 2011; Kamienkowski et al., 2012; Fischer et al., 2013; Trautmann-Lengsfeld et al., 2013; Simola et al., 2015). To remove corneoretinal potentials, which may have remained in the data even after eye blink correction using the pattern matching algorithm, and those related to saccadic eye movements, we added two regional sources with origins in the left and right eye orbit to the source dipole model. To demonstrate the capacity of the regional sources to control the artifact components caused by eye movements, we have quantified each subject’s residual variance in the individual average source dipole waveforms in two time intervals, one covering the onset of the trigger event (-15 – 40 ms) and the other corresponding to the interval showing statistically significant differences between objects and faces (170 – 210 ms). The source waveforms of three ECDs and effects of the presence of these regional sources on residual variance are illustrated in Figure 5.

In the first interval (-15–40 ms), the residual variance decreased from 70.1 ± 15% to 36 ± 20.7% (mean ± SD) after including two regional source dipoles into the source model. In the latency interval 170–210 ms, the residual variance changed from 41 to 27.5% after including two regional sources with origins in eye orbits. According to a two-way ANOVA for repeated measures (2 latency intervals, regional sources on vs. off), the addition of the two regional sources to the model showed a significant increase in the model fit for both latency intervals [*F*(1,18) = 152.7, *P* < 0.001]. The interaction between the latency intervals and presence of the regional sources in the source dipole model was also statistically significant [*F*(1,18) = 32.4, *P* < 0.001]. This interaction was caused by a stronger effect of the presence of two regional sources in the latency interval -15 – 40 ms compared to 170 – 210 ms.

The five-dipole model accounted for 83% of variance in the latency interval 0–300 ms. Attempts to fit further ECDs in subsequent latency intervals (>300 ms) did not reduce the residual variance significantly, and new ECDs landed outside the boundaries of the head. The slightly larger residual variance of 17% compared to laboratory studies achieving a residual variance 10% or smaller could be related to an increased background noise in our data which were recorded wirelessly in freely moving individuals and in the absence of control over incidental extraneous stimuli. Alternatively, this could be due to other active brain sources not accounted by the model.

### Source Dipole Waveforms in Face and Object Pictures

For each experimental condition the source dipole model was applied to the grand average data by projecting the source dipole model onto the original ERP data. The source waveforms of the three ECDs were analyzed using a 2 × 3 ANOVA for repeated measures over specific interval of interest from 0 to 300 ms. *P*-values were corrected using the false discovery rate method at *P* = 0.01.

**FIGURE 6:**
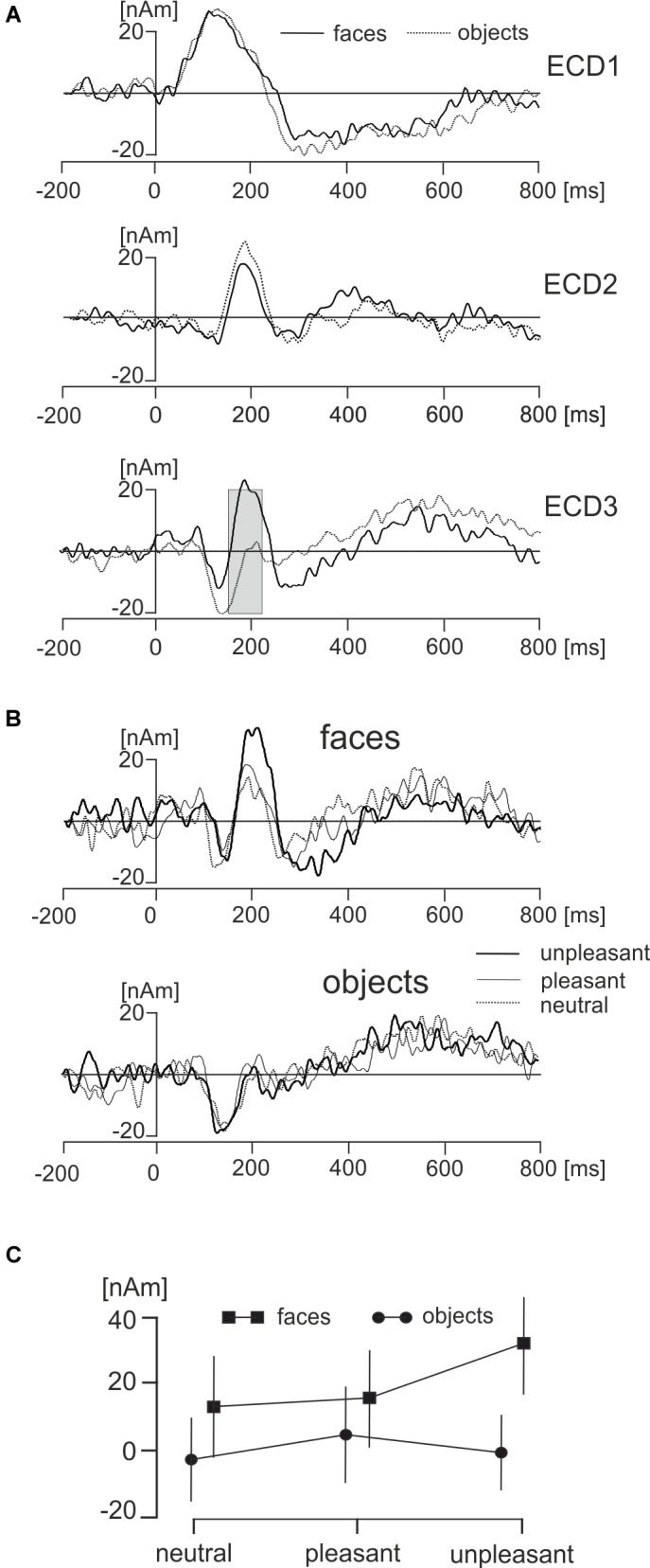
Effects of faces and objects on ECDs. **(A)** Mean ECD1–ECD3 source waveforms during viewing of faces (full line) and objects (dotted line). The gray rectangle in ECD3 indicates the interval 145–210 ms in which a statistically significant difference (corrected *P* < 0.01) between face and object pictures was found. **(B)** Mean ECD3 waveforms during viewing pleasant, unpleasant, and neutral faces and objects. **(C)** The statistically significant interaction between types of pictures (face, objects) and three hedonic valence levels (neutral, pleasant, and unpleasant).

The main effect representing the difference between face and object pictures was found only in ECD3 in a broad latency interval ranging from 145 to 210 ms (Figure 6A). The source dipole waveforms of ECD3 in faces and objects and in each of three hedonic levels are illustrated in Figure 6B. The latency interval showing a statistically significant difference between faces and objects comprised two local maxima in ECD3 source dipole waveforms. Face data showed a peak at 186 ms and objects data showed a peak at 211 ms. To analyze further the effects of picture types and three hedonic levels in the latency intervals showing the strongest activations in each of two types of pictures, the average source dipole moments in 10-ms intervals centered at the two peak latency points (181–191 ms and 196–216 ms) were analyzed using a 2 × 3 × 2 repeated measures ANOVA (2 picture types × 3 hedonic levels × 2 latency intervals). Faces showed a stronger source dipole amplitude than objects across both latency intervals [faces: 20.2 ± 4.5 nAm, objects: 0.56 ± 0.50 nAm, mean ± SD; *F*(1,18) = 29.3, *P* < 0.0001]. Further, the statistically significant interaction between picture types and three hedonic levels [*F*(1,18) = 9.86, *P* < 0.0001] revealed that the amplitude of ECD3 was affected by the hedonic content in faces but not in objects (Figure 6C). Tests of simple effects demonstrated the effect of hedonic levels were only significant in faces [*F*(2,36) = 15.9, *P* < 0.0001] but not in objects [*F*(2,36) = 1.58, *P* = 0.221]. The statistically significant effect of hedonic levels in face pictures was related to the greater amplitude of ECD3 in disgusted faces compared to both happy [*t*(18) = 15.95, *P* < 0.0001] and neutral faces [*t*(1,18) = 18.49, *P* < 0.0001].

**FIGURE 7:**
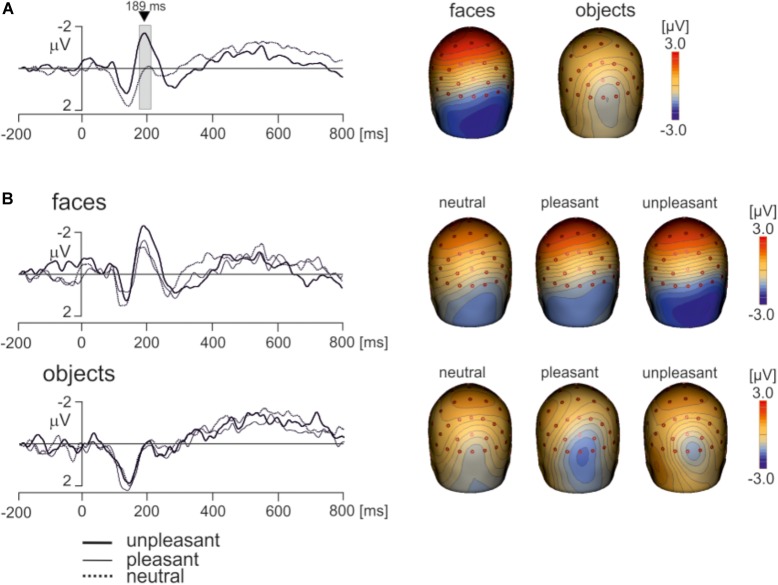
Grand average EMRP waveform for combined electrodes P8 and PO8. **(A)** Mean EMRP for all face and object conditions. The highlighted area represents the time window (175 – 212 ms) in which a statistically significant effect of picture type was found (*P* < 0.0001). **(B)** EMRPs for all three hedonic valence conditions for face and place images.

Electroencephalography data recorded using a wireless system in freely moving individuals are preferably interpreted based on source dipole analysis which allows to verify that a potential waveform of interest was of cerebral origin. However, we also analyzed if the differences between faces and objects and the effects of hedonic face valence shown in ECD3 would be present at select scalp electrodes. Two electrodes showing the face potential component at the latency of 189 ms, PO8 and P8 were averaged. The potential waveforms for faces and objects and the topographic maps of EMRPs at the latency of 189 ms are shown in Figure 7A. Figure 7B illustrates the PO8-P8 potential waveforms and topographic maps in neutral, pleasant and unpleasant faces and objects. The averaged PO8-P8 potential waveforms were analyzed using a 2 × 3 ANOVA for repeated measures over the interval from 0 to 300 ms. A statistically significant effect of picture type was found in the latency interval 175–212 ms [*F*(1,18) = 32.1, *P* < 0.0001]. The amplitude of the PO8-P8 potential was more negative in faces (-1.51 ± 0.40 mV) than in objects (0.32 ± 0.36 mV). In contrast to the ECD3 data, the interaction between picture types and three hedonic levels was not statistically significant [*F*(2,36) = 2.02, *P* = 0.16].

As our recordings did not allow to evaluate parameters of individual saccades which are known to affect the strength of P1 potential (Yagi, 1979; Thickbroom et al., 1991), we analyzed whether faces and objects would differ in the amplitudes of the P1 component. The potential in electrodes PO3 and PO4 showing maximum amplitudes of the P1 component were averaged. The amplitude of the P1 component in the interval 110–130 ms was analyzed statistically using a 2 × 3 ANOVA for repeated measures. Notably, the amplitude of the P1 component was almost identical in face and objects data [faces: 2.38 ± 0.26 mV, objects: 2.39 ± 0.36 mV (mean ± SEM); *F*(1,18) = 0.08, *P* = 0.95]. The P1 component was influenced neither by hedonic valence of pictures [*F*(2,36) = 0.41, *P* = 0.77] nor the interaction between the type of pictures and hedonic valence [*F*(2,36) = 1.86, *P* = 0.17]. It is therefore unlikely that the findings pertaining to the face-sensitive time interval (170–210 ms) would be affected either by low-level visual features of stimuli or differences in oculo-motor activity during viewing the pictures.

## Discussion

Recording brain electrical activity in natural settings during free visual exploration of the environment poses both technical and data analytical challenges. To the best of our knowledge, this study is the first to demonstrate the presence of a face-sensitive scalp potential during viewing of human faces in natural settings. The detection of the face sensitive potential in freely moving subjects using wireless EEG recordings could be accomplished owing to recent advances in EEG technology and data processing techniques. Our study uses an active electrode wireless EEG system which has been shown to cancel external electromagnetic noise (van Rijn et al., 1990). Unlike previous studies employing MoBI during walking or running (Gwin et al., 2010; Gramann et al., 2011; De Sanctis et al., 2012), head and body movement artifacts associated to the swaying of electrode cables were minimal in the present study because subjects stood calmly while viewing pictures. Furthermore, our gaze time analysis showed that all images, irrespective of their category and condition, were viewed for the same amount of time (approximately 3 s).

The N182 potential in our study showed a typical spatio-temporal pattern consistent with the N170 face potential occurring during viewing faces in laboratory type of EEG recordings, however, it was virtually absent during viewing objects. The face sensitive N182 component activity also differentiated disgusted and neutral faces evidencing that modern wireless mobile EEG recordings acquired in natural settings have the capacity to resolve emotional expressions of faces.

The face sensitive N182 component of EMRPs was modeled by an ECD located in the right fusiform area (Brodmann area 37) in the medial occipitotemporal cortex. The presence of a source in the fusiform gyrus is consistent with its role as a dominant face processing region (Yagi, 1981b; Kanwisher et al., 1997). Localizing the source of the N182 component in the fusiform gyrus is also consistent with previous source localization studies of face sensitive potentials in scalp EEG data (Bötzel et al., 1995; Deffke et al., 2007; Sadeh et al., 2010; Carretié et al., 2013; Trautmann-Lengsfeld et al., 2013) [reviewed in Rossion and Jacques (2011)] and in intracerebral, subdural (Allison et al., 1999) or depth electrode recordings (Barbeau et al., 2008).

ECD3 was localized to the fusiform area. The activity originating from this region showed a peak latency of 186 ms which falls within the broad latency limit of the N170 component ranging from 120 to 200 ms (Rossion, 2014). In contrast to face pictures, the source activity in the fusiform cortex was at a baseline level at the latency of 180–190 ms when subjects were viewing objects. This sharp contrast of the activity in fusiform cortex between viewing faces and objects strongly supports our conclusion that the N182 component of EMRP is equivalent to the face-sensitive N170 component in event-related potential recordings acquired in laboratory conditions.

One of the methodological challenges consisted in the separation of the lambda complex from corneoretinal artifacts of different origins. Eye movement artifacts are generated in most cases by rotations of the corneoretinal dipole crossing each eye predominantly in antero-posterior direction during eye blinks and saccades (Berg and Scherg, 1991; Dimigen et al., 2011; Nikolaev et al., 2016). The eye movement artifacts associated with eye blinking were removed at the pre-processing stage using a well-established pattern matching algorithm (Ille et al., 2002). To separate the genuine cortical potentials from the saccadic potentials, we employed a source dipole modeling approach. As saccadic eye movements associated with shifting the gaze toward different pictures had variable orientations, it was not possible to model the saccadic potentials using a set of equivalent current dipoles tuned to different directions of saccades (Berg and Scherg, 1991). Therefore, two sets of regional sources with fixed origins were placed to model the electrical dipoles resulting from the displacement of the eyeballs during saccadic movements in any direction. As each regional source had an identical spatial origin, the regional sources captured the saccade potentials irrespectively of the exact direction of a saccade at a particular instant. Further, employment of regional sources did not require modeling the residual corneoretinal or saccade potential at a specific latency period which allowed us to capture the components of the eye movement artifacts even in the presence of a slight latency jitter related to about 20 ms asynchrony between EEG and eye tracking data streams.

The EMRP waveforms in the present study featured a prominent lambda potential complex (Yagi, 1979, 1981b). The P1 component of lambda potential in the present study had a slightly longer peak latency of 120 ms compared to the 70–80 ms latency seen in earlier studies (Yagi, 1982; Thickbroom et al., 1991), but was close to the peak amplitude at a latency of 100 ms reported in more recent studies (Dimigen et al., 2011; Körner et al., 2014). These latency differences may be related to methodological and experimental variations across studies (Kamienkowski et al., 2012). For instance, the peaks of lambda potential have been shown to occur about 20 ms later if the time locking event is a saccade onset compared to setting the time-locking event to a fixation onset (Kaunitz et al., 2014).

The source activity generated in the medial occipitotemporal cortex also differentiated emotional expressions. Disgusted faces evoked stronger source activity than neutral or happy faces. Although earlier studies have questioned the capacity of the face sensitive N170 component to differentiate emotional expressions (Herrmann et al., 2002; Eimer et al., 2003), one recent study (Trautmann-Lengsfeld et al., 2013) and a recent meta-analysis involving 57 ERP studies involving a variety of emotional expressions showed that the face sensitive N170 component also differentiated emotional faces from neutral faces (Hinojosa et al., 2015). A comparatively strong amplitude modulation of the face-sensitive component by disgusted faces in the present study suggests that emotional and neutral face expressions are perceived differently while standing and behaving spontaneously. This may be related to participants standing upright in the present study as posture has been shown to affect a number of neurophysiological and cognitive parameters [reviewed in Thibault and Raz (2016)]. The specific role of posture on the face-sensitive ERP component and subjective ratings of different emotional expressions will be addressed in a future study. While this effect has been studied for auditory evoked components in the past (De Vos et al., 2014), this has yet to be done for emotional visual stimuli.

In contrast to previous laboratory studies of which some have found a modulation of mid- and long-latency event-related potential components by emotional expressions of faces (Eimer et al., 2003; Trautmann-Lengsfeld et al., 2013), our study did not identify any distinct evoked potential components in the latency epochs >300 ms. The absence of clear EMRP in the mid- and long-latency range may be limitation of wirelessly recorded data in freely behaving individuals. In contrast to laboratory studies, exploration of environment in the real world is a *trans*-saccadic process involving short-term visual memory, reframing, and prediction (Melcher and Colby, 2008). These higher order perceptual and cognitive processes continue during the exploration of a scene and since they would not show a fixed phase relative to the time-locking event, the resulting event-related potentials may not show any well-defined spatio-temporal components.

It should be pointed out that the face sensitive EMRP component analyzed in individual scalp electrodes did not resolve the hedonic valence of faces pictures. In the framework of mobile brain imaging (Gramann et al., 2010; Gwin et al., 2010, 2011), source dipole localization is an important element in the data processing pipeline because it allows for separation of cerebral and extracerebral contributions to scalp potentials. In the present study, source dipole localization separated the occipital cortex activations associated with lambda potential from the face sensitive N182 component. Therefore, source waveform data were more specific to face processing and extracted the face sensitive activation better than scalp electrode data. The methodological feature of wirelessly recorded EEG data in freely moving individuals needs to be taken into account when comparing our data with previous lab-based studies of the N170 potential.

The difference between the current experiment and previous research may be related to the fact that, in contrast to previous studies where subjects were seated and passively viewing a computer monitor, the subjects in our study were standing and able to freely control their movements. The effects of body posture and freedom of making simple behavioral decisions on emotional expressions is still poorly understood. Humans may perceive different levels of various coping resources while standing compared to sitting or reclining. For instance leaning forward compared to reclining has been shown to shorten reaction times and increase the late cortical positive potential for appetitive cues (Price et al., 2012). Since an appraisal of coping resources contributes to the perception of a situation as taxing or stressful (Lazarus and Folkman, 1984), it is likely that emotional responses and their cortical representations may have different characteristics in people while moving and behaving freely compared to when they are seated and restrained by a laboratory setup. The finding of stronger anticipatory anxiety before a stressful mental arithmetic task during standing than supine (Lipnicki and Byrne, 2008) suggests that emotional processing of the same stimuli may vary depending on the body posture. Future studies should more carefully address the effects of body posture on emotional and cognitive processes, including face processing; as future findings acquired using mobile EEG recordings in natural settings will likely differ from those acquired in laboratory conditions.

To conclude, we showed that EMRPs acquired using combined eye-tracking and wireless EEG recordings in freely moving individuals clearly differentiated between viewing a human face and a non-living object as well as between types of emotional face expression. These findings open new questions, for instance the effect of posture on naturally occurring ERPs. The methodology presented provides a range of experimental and applied research possibilities in multiple domains including social and developmental psychology, medicine, and consumer science.

## Data Availability

The datasets generated during and/or analyzed during the current study are available from the corresponding author on reasonable request.

## Author Contributions

AS contributed the original concept behind this work. VS carried out the data acquisition and pre-processing of the collected data. JT-C, KK, HR, and SC assisted in collecting data for this experiment. VS and AS contributed to the production of the final written manuscript including data analysis and figure production. AS created the original computer programs for data analysis and synchronization of mobile EEG/eye-tracking data. VS, AS, NF, and TG contributed to the experimental design as well as the large-scale planning of this project. AS, NF, and TG secured funding for project.

## Conflict of Interest Statement

The authors declare that the research was conducted in the absence of any commercial or financial relationships that could be construed as a potential conflict of interest.

## Supplementary Information

Any supplementary material (i.e., panel stimuli, raw eye-tracking data, and raw EEG data) for the current study will be available from the corresponding author upon reasonable request for a period of no less than 5 years.
